# Zbtb1 prevents default myeloid differentiation of lymphoid-primed multipotent progenitors

**DOI:** 10.18632/oncotarget.11356

**Published:** 2016-08-17

**Authors:** Xianyu Zhang, Ying Lu, Xin Cao, Tao Zhen, Damian Kovalovsky

**Affiliations:** ^1^ Experimental Immunology Branch, NCI, NIH, Maryland, USA; ^2^ College of Life Science and Engineering, Northwest University for Nationalities, Gansu Engineering Research Center for Animal Cell, Lanzhou, China; ^3^ Oncogenesis and Development Section, National Human Genome Research Institute, NIH, Maryland, USA

**Keywords:** Zbtb1, lymphoid, development, myeloid, differentiation, Immunology and Microbiology Section, Immune response, Immunity

## Abstract

Zbtb1 is a transcription factor that prevents DNA damage and p53-mediated apoptosis in replicating immune progenitors, affecting lymphoid as well as myeloid development when hematopoietic progenitors are in competition in mixed bone marrow chimeras. However, Zbtb1-deficient mice do not have an apparent myeloid deficiency. We report here that Zbtb1-deficient lymphoid-primed multipotent progenitors (LMPPs) are biased to develop towards the myeloid fate in detriment of lymphoid development, contributing to the apparent unaffected myeloid development. Zbtb1 expression was maintained during lymphoid development of LMPP cells but downregulated during myeloid development. Deficiency of Zbtb1 in LMPP cells was sufficient to direct a myeloid fate in lymphoid-inducing conditions and in the absence of myeloid cytokines as shown by upregulation of a myeloid gene signature and the generation of myeloid cells *in vitro*. Finally, biased myeloid differentiation of Zbtb1-deficient LMPP cells was not due to increased p53-dependent apoptosis as it was not reverted by transgenic Bcl2 expression or p53 deficiency. Altogether, our results show that Zbtb1 expression prevents activation of a default myeloid program in LMPP cells, ensuring the generation of lymphoid cells.

## INTRODUCTION

Many members of the BTB-ZF (Broad complex, tramtrack, and Bric a brac-zinc finger) family of transcription repressors were shown to play key roles in the development of immune cells [1, 2]. Zbtb1 is a recently identified member of this family, which is essential for the development of T-cells. A point mutation in the BTB domain of Zbtb1 termed “ScanT”, as well as Zbtb1 deficiency, leads to complete absence of T-cells [3, 4]. In bone marrow chimeras, however, Zbtb1-deficiency leads to a more profound immune developmental defect, completely ablating the generation of lymphoid cells and partially affecting the generation of myeloid cells [5]. Impaired immune cell development is consequence of increased DNA damage, leading to p53-mediated apoptosis in uncommitted immune progenitors that undergo rapid proliferation.

During proliferation, absence of factors required for DNA synthesis or mutations in the DNA that lead to stalling of the replication fork initiate a replication stress response to repair the DNA lesion before mitosis, preventing double strand breaks in the DNA. During replication stress, Zbtb1 interacts with Kap1, allowing the accessibility of Rad18 to sites of replication stress, which in turn triggers the recruitment of low fidelity DNA polymerases that can bypass the DNA lesion ensuring continuation of DNA synthesis. This process is called translesion DNA synthesis [6].

Replication stress and accumulated DNA damage drives the functional decline of HSCs with age [7]. Aged HSCs are impaired to reconstitute the immune system due to increased senescence and apoptosis [8, 9], have lost lymphoid potential and preferentially generate myeloid cells [10]. As increased DNA damage and activation of the p53-p21 axis were linked to biased myeloid development of hematopoietic stem cells [11, 12], and as Zbtb1 was shown to prevent DNA damage, we evaluated if Zbtb1-deficient immune progenitors presented biased myeloid development.

Here we identified that Zbtb1 expression was reduced during myeloid but not lymphoid differentiation and absence of Zbtb1 led to activation of a default myeloid program in lymphoid-primed multipotent progenitors (LMPP), and to the generation of myeloid cells in the presence of lymphoid cytokines and absence of myeloid cytokines. Unexpectedly, however, this phenotype was not related to increased apoptosis or activation of the p53 cell stress pathway as it could not be reverted by either transgenic bcl2 expression or p53-deficiency.

In summary, our results show that Zbtb1 ensures lymphoid development by preventing a default myeloid program in LMPP cells.

## RESULTS

### Zbtb1 deficiency leads to biased myeloid development

**Figure 1:**
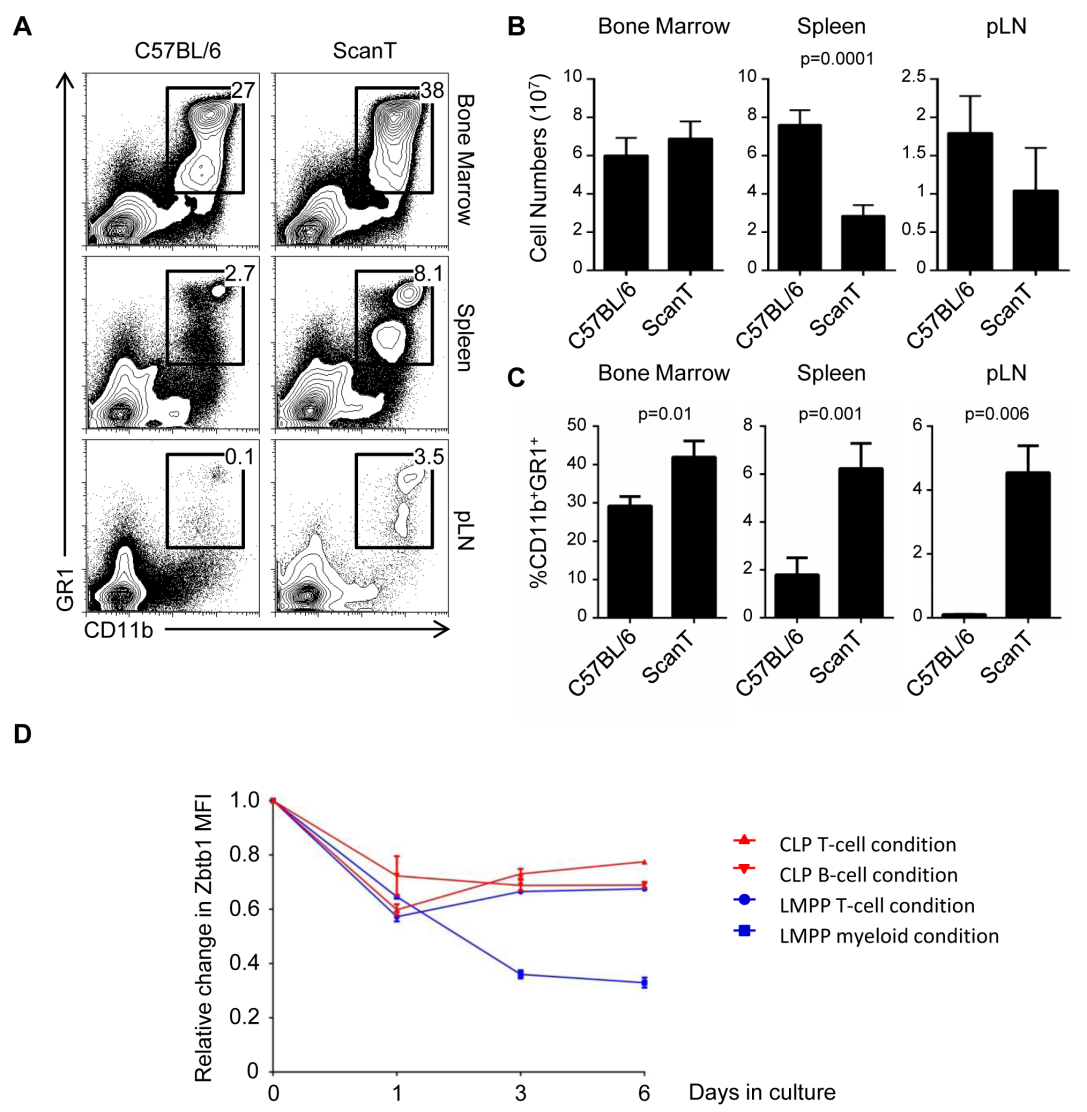
ScanT mice have a proportionally increased myeloid compartment **A.** FACs analysis of cell suspensions isolated from the indicated tissues. The numbers correspond to the proportion of events within the gates. **B.** Analysis of total cell numbers in the indicated tissues. **C.** Analysis of the proportion of CD11b^+^GR1^+^ obtained from the indicated tissues. **D.** Analysis of relative change in GFP levels from Zbtb1-GFP reporter mice. GFP MFI was measured in the indicated conditions before and after culture of cells in OP9-DL1 (T-cell condition); OP9 (B-cell condition) or OP9 plus myeloid cytokines (myeloid condition) for the indicated times. Represented data corresponds to the relative change MFI of Zbtb1-GFP/MFI of C57BL/6 at the indicated times. Data is representative of more than 3 independent experiments. p values corresponding to the significance using *T*-test are shown.

Zbtb1-deficient mice have impaired lymphoid but not myeloid development [3, 4]. However, we have observed that under competitive conditions, both myeloid and lymphoid development were affected due to activation of p53-dependent apoptosis before commitment to either lymphoid or myeloid lineages [5]. We therefore postulated that compensatory mechanisms must exists that increase myeloid development and counterbalance this general deficiency in uncommitted immune progenitors. To evaluate this, we analyzed the proportion of myeloid cells in wild type and ScanT (Zbtb1-mutant) mice, which lacks Zbtb1 protein [5]. We observed that ScanT mice have a significantly increased proportion of myeloid (CD11b^+^GR1^+^) cells in bone marrow, spleen and lymph nodes (Figure 1A). However, this increased proportion didn't correlate with an increase in myeloid cell numbers due to the lower total cellularity in the spleen and lymph nodes of ScanT mice (Figure 1B and 1C).

### Zbtb1 expression is downregulated during myeloid development

Hematopoietic stem cells and immune progenitors express high levels of Zbtb1 [3, 4]. We have also reported that granulocytes express very low levels of Zbtb1 [5]. We therefore postulated that Zbtb1 expression might be maintained during lymphoid development but downregulated during myeloid development. To evaluate this, we isolated lymphoid-primed multipotent progenitors (LMPP) or common lymphoid progenitors (CLP) from Zbtb1-GFP reporter mice and induced T-cell or B-cell development *in vitro* by co-culture with OP9-DL1 or OP9 stroma cells respectively in the presence of lymphoid cytokines (IL-7, Flt3L) or myeloid development by culture with myeloid cytokines (GM-CSF, M-CSF, IL-3). We observed that Zbtb1 expression was maintained during lymphoid development but it was downregulated during myeloid development (Figure 1D), opening the possibility that downregulation of Zbtb1 may have a causal effect on myeloid development.

### Zbtb1 represses myeloid development in LMPP cells

**Figure 2:**
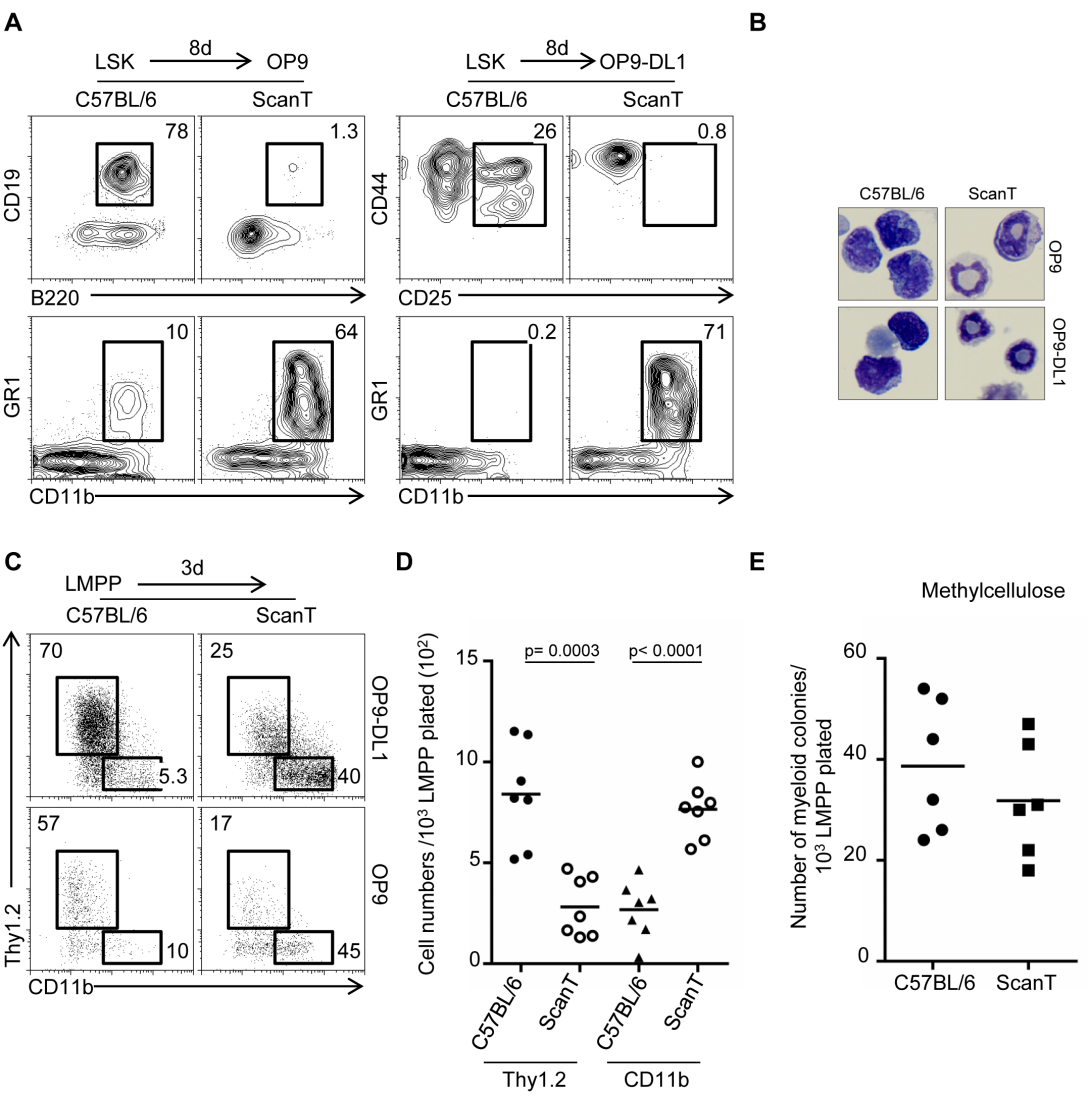
Zbtb1 prevents a default myeloid differentiation of LMPP in lymphoid inducing conditions **A.** FACS analysis of cells obtained after co-cultured with OP9 or OP9-DL1 stromal cells in the presence of lymphoid (IL-7 and Flt3l) and absence of myeloid cytokines for 8 days to initiate lymphoid development. The numbers indicate the proportion of cells obtained within the gates. **B.** Representative Giemsa staining showing the morphology of cells obtained in the co-cultures represented in (A). **C.** FACs analysis of cells obtained after a 3-day co-culture of sorted LMPP cells. The numbers indicate the proportion of cells obtained within the gates. **D.** Gram plot showing the number of cells obtained during the co-cultures represented in (C). Each dot corresponds to data from a mouse. Horizontal bars represent the mean. **E.** Gram plot showing the number of myeloid colonies obtained after culture of sorted LMPP cells in methylcellulose in the presence of myeloid cytokines. Each dot corresponds to data from a mouse.

To evaluate if downregulation of Zbtb1 controls commitment towards the myeloid fate in immune progenitors, we analyzed the potential of wild type and ScanT LSK (lin-Sca1^+^ckit^+^) cells to initiate lymphoid and myeloid differentiation under lymphoid-inducing conditions and in the absence of myeloid cytokines, conditions that do not normally support myeloid differentiation. We observed that ScanT progenitors failed to develop into B-cells and T-cells by co-culture with OP9 and OP9-DL1 stroma cells respectively, as previously described [5]. Interestingly, ScanT but not wild type progenitors acquired a myeloid phenotype (CD11b^+^GR1^+^) in these cultures (Figure 2A). Giemsa analysis of the cells obtained after culture showed that wild type cells have a mononuclear morphology characteristic of lymphoid cells and ScanT cells had a polynuclear morphology characteristic of neutrophils and immature myeloid cells (Figure 2B).

We then evaluated if ScanT LMPP cells were biased to differentiate into myeloid cells in lymphoid-inducing conditions. For this experiment we choose a short-term culture of three days because myeloid cells become apoptotic in the absence of myeloid cytokines under longer culture conditions. Under these conditions, approximately 60-70% of wild type LMPP cells acquired a lymphoid (Thy1.2+) phenotype and only 5-10% of cells generated myeloid (CD11b^+^) cells. Interestingly, approximately 40-45% of ScanT LMPP cells became myeloid (CD11b^+^) cells. Biased myeloid development was independent of Notch signals as it similarly occurred by co-culture with stroma cells expressing (OP9-Dl1) and not expressing (OP9) the Notch ligand delta like 1 (DL1) (Figure 2C). This proportional increase of myeloid cells from ScanT progenitors was not due to the specific apoptosis of cells that initiated the T-cell or lymphoid program as ScanT progenitors generated 2.5-fold more myeloid cell numbers than wild type progenitors (Figure 2D). Thus, ScanT LMPP cells generate myeloid cells in lymphoid-promoting conditions.

One possible cause for the biased myeloid differentiation of ScanT progenitors could be that ScanT LMPP cells are heterogeneous with more cells able to generate myeloid cells (myeloid potential) than wild type LMPP cells. We tested this by performing cultures of sorted LMPP cells in semisolid media (methylcellulose) in the presence of myeloid cytokines for six days. Under these myeloid-inducing conditions, a single LMPP progenitor, if it has the potential to develop into a myeloid cell, generates a myeloid colony, therefore, the number of myeloid colonies generated represents the number of LMPP cells that had the potential to become myeloid cells. Interestingly, there were no significant differences in the number of myeloid colonies generated from wild-type and ScanT LMPP cells (Figure 2E), indicating that ScanT LMPP cells have the same myeloid potential as wild type cells. We have previously described that ScanT LSKs didn't present an increase of myeloid-biased (CD150^+^) HSC cells [5], in correlation with the similar myeloid potential of ScanT progenitors.

In conclusion, our results indicate that Zbtb1 does not affect the myeloid potential of immune progenitors but it is required for immune progenitors to initiate lymphoid commitment, as absence of functional Zbtb1 re-directs lymphoid progenitors into a myeloid fate.

### Zbtb1 prevents the spontaneous activation of the myeloid program in LMPP progenitors

**Figure 3:**
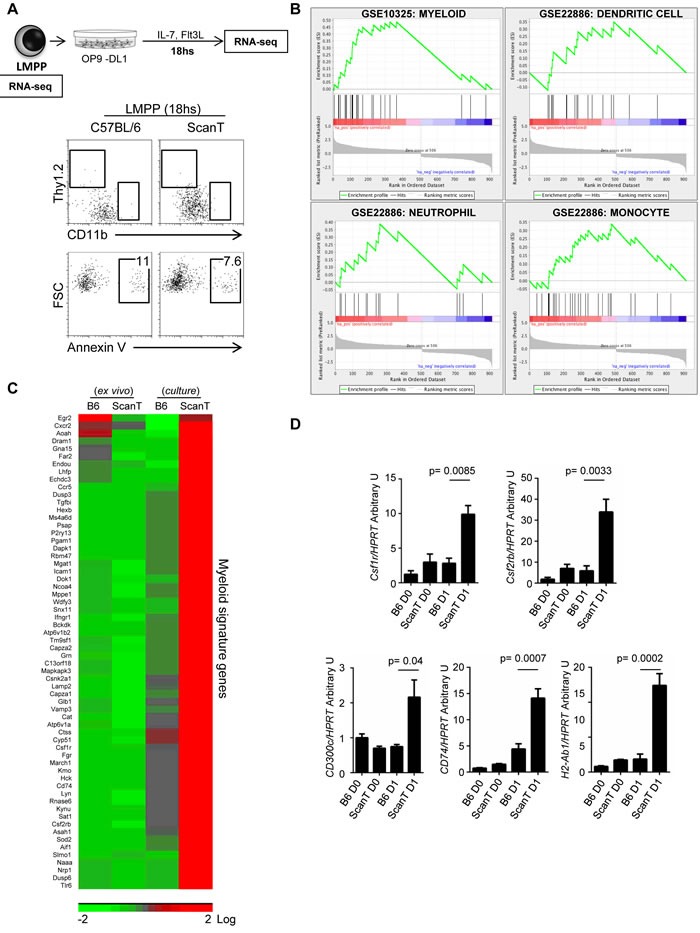
Zbtb1 represses the myeloid program in LMPP cells **A.** Diagram showing the cell samples LMPP cells ex-vivo and after 18hs culture with OP9-DL1 in lymphoid inducing conditions, which were used for RNAseq assays. FACS analysis showing the phenotype of LMPP cells and percentage of annexin V+ cells after OP9-DL1 co-culture. The numbers indicate the proportion of cells within the gate. **B.** Gene Set Enrichment Analysis (GSEA) of pre-sorted genes showing differential expression between wild type and ScanT cells. The plots represent the gene enrichment observed in ScanT LMPP cells for genes expressed in the indicated immune subsets. **C.** Heatmap of selected myeloid signature genes represented in (B) showing the expression value in LMPP cells *ex vivo* and after culture. **D.** RT-PCR of selected genes to validate the RNAseq data. Data is representative of 3 independent experiments. p values corresponding to the significance using *T*-test are shown.

In order to understand how Zbtb1 affects lymphoid commitment, we sorted LMPP cells from wild type and ScanT bone marrow and performed RNA-seq directly *ex-vivo* or after 18hs incubation in OP9-DL1 co-cultures in the presence of lymphoid (IL-7 + Flt3l) and absence of myeloid cytokines to initiate the T-cell developmental program. We choose this short incubation time as we identified that LMPP cells have not yet upregulated markers of differentiation into either lymphoid (Thy1.2^+^) or myeloid (CD11b+) lineages and were not apoptotic (Figure 3A). Analysis of gene expression between wild type and scanT LMPP cells after culture identified 916 unique gene identifiers that were significantly upregulated or downregulated in ScanT cells. This pre-ranked gene list was used for gene set enrichment analysis (GSEA) against the immunological signature to identify if the differential expression pattern correlated with publicly available gene expression datasets. This analysis revealed enrichment of a myeloid transcriptional signature in ScanT cells, including neutrophils, monocytes, and dendritic cells (Figure 3B). This transcriptional signature corresponded to 62 unique genes that were not expressed in ScanT or wild type cells ex vivo and were upregulated after culture in ScanT cells and not in wild type cells (Figure 3C). We corroborated that ScanT LMPP and not wild type LMPP cells initiated the myeloid program after culture by RT-PCR for some identified markers (Figure 3D). Interestingly, we found a similar expression pattern on *Csf1r* (encoding M-CSFR) and *Csf2rb* (encoding the common subunit of GM-CSFR and IL-3), which not only are markers of myeloid cells but also direct myeloid differentiation of HSCs in response to myeloid cytokines [13-16].

These results show that ScanT LMPP cells, despite having a similar myeloid potential than wild type cells, differentiate into myeloid cells when cultured in lymphoid inducing conditions, indicating that Zbtb1 is required to repress the myeloid program in LMPP progenitors.

### Increased p53-mediated apoptosis is not the cause of the myeloid bias of ScanT LMPP cells

**Figure 4:**
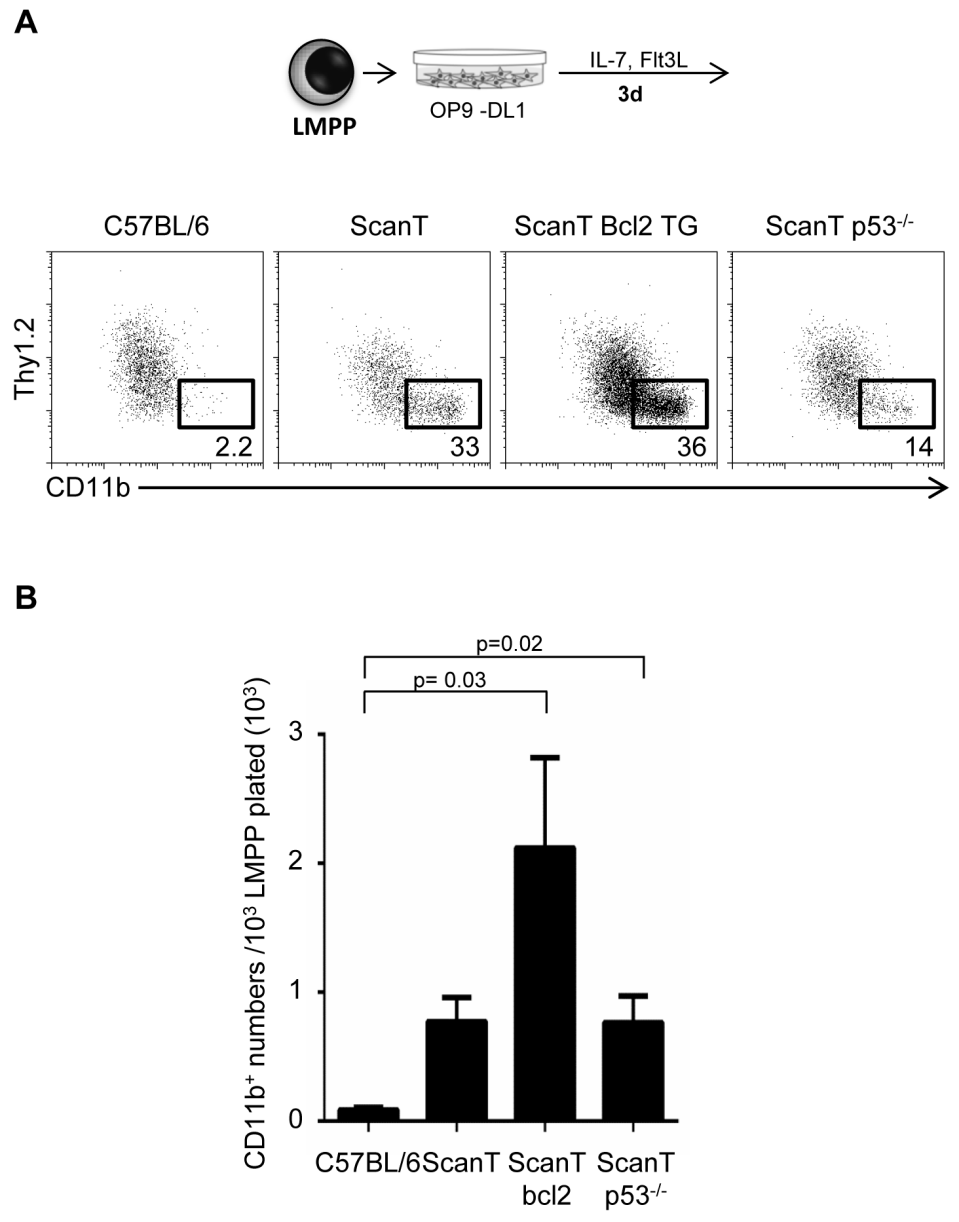
Protection from apoptosis or p53-deficiency does not rescue the myeloid bias of ScanT LMPP cells **A.** FACs analysis of cells obtained after a 3-day co-culture of sorted LMPP cells with OP9-DL1 cells. The numbers indicate the proportion of cells obtained within the gates. **B.** Analysis of myeloid (CD11b^+^) cell numbers obtained after culture. Data is representative of 3 independent experiments. p values corresponding to the significance using *T*-test are shown.

We have previously identified that ScanT progenitors undergo increased DNA damage and p53-dependent apoptosis [5]. As increased p21 levels and cell cycle lengthening that may occur after activation of p53 in response to DNA damage and be causal of myeloid differentiation [11, 12], we decided to evaluate if bcl2 transgenic expression, which protects cells from apoptosis [17] and p53-deficiency would revert the myeloid bias of ScanT progenitors. We tested this by analyzing the acquisition of myeloid and lymphoid markers in sorted LMPP cells after OP9-DL1 co-culture in lymphoid conditions. Interestingly, ScanT x Vav-Bcl2 and ScanT x p53−/− LMPP cells generated more myeloid cells than wild type LMPP cells, similarly to ScanT progenitors (Figure 4A and 4B). Therefore, activation of p53-mediated apoptosis is not causative of the myeloid bias of ScanT progenitors.

Altogether, we have observed that Zbtb1 is downregulated during myeloid differentiation of LMPP cells and further showed that Zbtb1 deficiency is sufficient to direct the myeloid program in the absence of myeloid inducing conditions. Our data shows a requirement of Zbtb1 to prevent default myeloid development of lymphoid-primed progenitors strengthening the role of Zbtb1 as a safeguard of lymphoid commitment and differentiation.

## DISCUSSION

Myeloid commitment occurs in multipotent pluripotent progenitors by the action of cytokines and transcription factors. Low expression levels of M-CSFR (*Csf1r*) in immune progenitors allow these cells to signal to M-CSF to instruct lineage choice towards the myeloid fate [16]. Enforced expression of GM-CSFR (*Csf2rb/Csf2ra*) in common lymphoid progenitors (CLP) and pro-T cells is also sufficient to direct granulocyte macrophage differentiation in committed lymphoid progenitors [13]. Therefore, signals through M-CSFR and GM-CSFR appear sufficient to direct myeloid fate. Our results show that Zbtb1-deficiency in LMPP cells is sufficient to direct myeloid differentiation in lymphoid inducing conditions and this fate re-direction correlated with increased expression levels of *Csf1r* and *Csfr2b*.

Two major transcription factors have been implicated in myeloid fate. PU.1, encoded by *spi1*, is required for HSC maintenance [18], and higher PU.1 levels direct myeloid development, while lower levels are required for B-cell development [19]. PU.1 induces *Csf1r* providing a positive loop towards myeloid differentiation [20]. C/EBPα is also sufficient to drive the development of macrophages from pre-T-cells [21] and granulocyte-macrophages from CLP cells [22]. However, and surprisingly we didn't detect increased expression levels of *Spi1* or *Cebpa* in ScanT progenitors (data not shown).

Replication stress and accumulated DNA damage underlie the functional decline of HSC cells with age [7, 23]. Aged HSCs are impaired to reconstitute the immune system, which has been associated with increased senescence and apoptosis [24, 25]. Notably though, aged HSCs have lost lymphoid potential and preferentially differentiate into myeloid cells. We have observed that, in correlation with increased DNA damage in ScanT hematopoietic progenitors [5], ScanT hematopoietic progenitors have lost lymphoid potential and preferentially developed into myeloid cells. This biased myeloid differentiation was consequence of default activation of the myeloid program in the absence of myeloid cytokines.

A possible mechanism to explain the biased myeloid development of aged HSCs is that HSCs are heterogeneous and that a subset of myeloid-biased HSCs amplify in older mice [26]. These myeloid-biased HSCs can be identified as CD150^hi^CD41^+^ LT-HSC [27]. We have previously analyzed this subset and have not observed amplification of CD150^+^ LT-HSCs in ScanT mice [5]. Our results are in agreement with those reported in MLL4-deficient mice. MLL4-deficient HSCs develop into myeloid cells due to increased oxidative stress and DNA damage in HSCs. Similar to ScanT mice, MLL4-deficient mice didn't present an increase of myeloid-biased LT-HSCs [12]. We have also observed that ScanT LMPP cells had the same myeloid potential as wild type cells as they generate a similar number of myeloid colonies in methylcellulose cultures in the presence of myeloid cytokines.

Another proposed mechanism by which increased DNA damage may lead to myeloid bias is that increased p21 levels downstream activation of p53 leads to lengthening of the cell cycle and lack of dilution of PU.1 protein levels. Therefore, if PU.1 levels are not diluted by rapid proliferation, higher PU.1 levels may lead to myeloid commitment of immune progenitors [8, 11, 18]. Based on our data, this mechanism is however unlikely because of several reasons: First, the myeloid bias of ScanT LMPP cells was observed already at 18 hours post-culture, a time frame in which extensive proliferation have not yet occurred; second, we have previously reported that ScanT cells didn't show a defect in proliferation [5]; third, p53-deficiency,which should prevent a p21-dependent cell cycle arrest, didn't rescue the myeloid bias of ScanT progenitors.

Altogether, our results better support a model in which Zbtb1 actively represses the myeloid program in lymphoid progenitors without which cells are poised to develop into myeloid cells. Future work identifying the DNA targets of Zbtb1 and how absence of Zbtb1 alters the chromatin structure of hematopoietic progenitors will provide insights into its mechanism of action.

## MATERIALS AND METHODS

### Flow cytometry and cell sorting

Single-cell suspensions were prepared at the time of autopsy from bone marrow, lymph nodes or spleen in PBS containing 0.1% BSA and 0.08% sodium azide (staining buffer). Antibody incubation was performed at 4 °C for 20 min in staining buffer. For LMPPs and CLPs, freshly isolated bone marrow cells were incubated with biotinylated antibodies to CD3ε (145-2C11), B220 (RA3-6B2), TER-119 (TER-119), CD11b (M1/70), Gr-1 (RB6-8C5). Lineage-positive (Lin^+^) cells were depleted by MACS separation system according to manufacturer's recommendations. After enrichment Lin- cells were stained with surface markers. LMPPs were sorted by flow cytometry as Lin-Sca-1^+^c-Kit+Flt3^hi^, and CLPs were sorted as Lin-IL-7Rα^+^AA4.1^+^Flt3^+^. Antibodies used for flow cytometry were listed below:

CD45.1 (A20), CD45.2 (104), Sca-1 (E13-161.7), c-Kit (2B8), Flt3 (A2F10), IL-7Rα (A7R34), CD93 (AA4.1), B220 (RA3-6B2), CD19 (6D5), CD11b (M1/70), Gr-1 (RB6-8C5), CD44 (IM7), CD25 (PC61), Thy1.2 (30-H12), CD4 (RM4-5), CD8α (53-6.7), TCR-β (H57), P53 (1C12).

Analysis of FACS data was done using the Flojo software.

### Cell culture

To induce the differentiation of hematopoietic progenitors LSK (Lin-Sca1+ckit+), LMPP (LSK Flt3hi) or CLP (Lin-IL-7Rα^+^AA4.1^+^Flt3^+^) cells into B or T-cells *in vitro*, bone marrow sorted cells were co-cultured with OP9 and OP9-DL1 stroma cells respectively in αMEM medium supplemented with 5 ng/ml Flt3L and 1 ng/ml IL-7, 10%FBS, antibiotics and β-mercaptoethanol. OP9 culture for myeloid differentiation was performed in αMEM supplemented with 2.5 ng/ml SCF, 7.5 ng/ml Flt3L, 0.5 ng/ml IL-3, 0.5 ng/ml M-CSF, 0.5 ng/ml GM-CSF, 1 ng/ml G-CSF, 1 ng/ml IL-7.

### Annexin staining

Staining procedure was performed according to the manufacturer instructions (eBioscience). After cell surfaces' staining, cells were washed in PBS and resuspended in 1×annexin binding buffer containing FITC-conjugated annexin V. After incubation at room temperature for 15 min, cells were diluted in 1×annexin V binding buffer and analyzed by flow cytometry.

### *In vitro* labeling and staining of BrdU

Dividing Cells were *in vitro* labeled with 10 μM BrdU for 30 min at 37 °C. Stain with Fixable Viability Dye (FVD) to label dead cells before fixation. Stain cell surface antigens. Cells were fixed with freshly prepared 1×BrudU Staining Buffer work solution for 15 min at room temperature in the dark. After wash and resuspension cells were digested with DNase I working solution for 1 hour at 37 °C in the dark. Stain with anti-BrdU antibody for 20-30 min at room temperature in the dark.

### Modified giemsa staining

Cytospins were stained by Differential Quik Stain kit (Polysciences, Inc.) according to manufacturer's instruction.

### Mouse colony-forming cell (CFC) assays using methocult®

LMPPs were suspended in αMEM containing 25 ng/ml SCF, 75 ng/ml Flt3L, 5 ng/ml IL-3, 5 ng/ml M-CSF, 5 ng/ml GM-CSF, 10 ng/ml G-CSF, 10 ng/ml IL-7. Cells were combined with MethoCult® at a ratio of 1:10 (vol/vol). Mix thoroughly and dispense the MethoCult® mixture containing cells into 35 mm dish. Incubate at 37 °C, in 5 % CO_2_ with ≥95 % humidity for 12 days. Colonies were observed under phase contrast microscope.

### RNA isolation and real time PCR

RNA was extracted using RNeasy Plus Micro Kit (QIAGEN) and reverse transcribed using iScript™ cDNA Synthesis Kit (BIO-RAD). Real time PCR was performed in a 10 μl reaction volume with iTaq™ Universal SYBR® Green Supermix (BIO-RAD). Reaction was performed on 7900HT Fast Real-Time PCR System (Applied Biosystems). The expression of the gene of interest was calculated relative to the HPRT mRNA. Primers uses for RT-qPCR are listed below.

Csf1r forward, 5′-TGTCATCGAGCCTAGTGGC-3′

Csf1r Reverse, 5′-CGGGAGATTCAGGGTCCAAG-3′

Csf2rb2 forward, 5′- TCCAGCCAGATCGTGACCT-3′

Csf2rb2 reverse, 5′-AATCCCCAAGAGATACACTCCA-3′

CD300C forward, 5′- TGAGGTGTTCGTGGTCCCA-3′

CD300C reverse, 5′- CAGAATGTTGATACCGTTCCCA-3′

CD74 forward, 5′- AGTGCGACGAGAACGGTAAC-3′

CD74 reverse, 5′- CGTTGGGGAACACACACCA-3′

H2-Ab1 forward, 5′- TGAACAGCCCAATGTCGTCAT-3′

H2-Ab1 reverse, 5′- CAGCGCACTTTGATCTTGGC-3′

HPRT forward, 5′-TCAGTCAACGGGGGACATAAA-3′

HPRT reverse, 5′-GGGGCTGTACTGCTTAACCAG-3′

### RNA-Seq and GSEA analysis

To perform RNAseq analysis, RNA was isolated from sorted cells and cDNA was amplified using the SMARTer ultra low input amplification kit. Generated libraries were sequenced in HiSeq2500 as pooled samples.

Bioinformatic analysis of the RNAseq data was performed by the CCR bioinformatic core. Briefly Raw fastq files for each of the samples are evaluated with FastQC for quality and adapter contamination; the adapters and low quality bases are trimmed with Trimmomatic software. The trimmed fastq files are aligned to the mm10 reference genome using STAR v2.4.0a algorithm to generate BAM files, and these aligned BAM files are then taken through HTSeq v0.6.1 to do read counting. The read counts are then normalized by DESeq2 v3.1 using a scaling factor method that is applied across all samples for all genes, and then analyzed for differential expression profiles. Statistical filters applied (*p*-val < 0.05 and AbsFold-Change1.5) to generate specific lists of differentially expressed genes for each group comparison.

The differential expression analysis of RNAseq data for the contrast groups WT *vs* ScanT within D1 of the LMPP samples were used as a pre-ranked input list for GSEA. This input list comprised of 916 genes and was ranked according to descending fold-change. GSEA Pre-ranked analysis was run on the Immunological Signature (C7 of MSigDB) to query for enriched gene sets, with a specific focus on sets that were related to T cells, B cells, Myeloid progenitors, monocyte, macrophages and dendritic cells. All of the four enriched gene sets reported here have a significant *p* value < = 0.09, and an FDR q—val < 0.2 (the first two have FDR q—val < 0.005) There are 62 unique genes displayed as being enriched across these 4 datasets. These 62 genes are used to plot the heatmap for the 4 sample groups in WT and ScanT.

For the construction of the heatmap, the complete matrix of normalized count data for all samples generated from DESeq2 was imported onto Partek Genome Studio v6.6. The relative expression values for the custom gene list to be plotted on the heatmap were extracted for the specific sample subsets to be displayed on the figure. Hierarchical clustering with euclidean distances was used to generate the

GSEA analysis: The differential expression analysis of RNASeq data for the contrast groups WT vs ScanT within D1 of the LMPP samples were used as a Preranked input list for GSEA. This input list comprised of 916 genes and was ranked according to descending fold—change. GSEA Preranked analysis was run on the Immunological Signature (C7 of MSigDB) to query for enriched gene sets, with a specific focus on sets that were related to T cells, B cells, Myeloid progenitors, monocyte, macrophages and dendritic cells. All of the four enriched gene sets reported here have a significant *p* < = 0.09, and an FDR *q* < 0.2 (the first two have FDR *q* < 0.005) There are 62 unique genes displayed as being enriched across these 4 datasets. These 62 genes are used to plot the heatmap for the 4 sample groups, LMPP D0 and D1 for WT and ScanT.
